# Supplementary material for: Modulating glycosphingolipid metabolism and autophagy improves outcomes in pre-clinical models of myeloma bone disease
Source: Nat Commun. 2022 Dec 22;13:7868. doi: 10.1038/s41467-022-35358-3 (PMC9780346; doi:10.1038/s41467-022-35358-3)

Supplementary Materials for

Modulating glycosphingolipid metabolism and autophagy improves outcomes in pre-clinical models of myeloma bone disease

Houfu Leng, Hanlin Zhang, Linsen Li, Shuhao Zhang, Yanping Wang, Selina J Chavda, Daria Galas-Filipowicz, Hantao Lou, Adel Ersek, Emma Morris, Erdinc Sezgin, Yi-Hsuan Lee, Yunsen Li, Ana Victoria Lechuga-Vieco, Mei Tian, Jianqing Mi, Kwee Yong, Qing Zhong, Claire M Edwards, Anna Katharina Simon*, Nicole J. Horwood*.

* To whom correspondence should be addressed:

Prof.Katja Simon: katja.simon@imm.ox.ac.uk; Prof.Nicole Horwood: n.horwood@uea.ac.uk

Table of Contents

SUPPLEMENTARY TABLE **2**

SUPPLEMENTARY TABLE 1: BASELINE MM PATIENT CHARACTERISTICS 2
SUPPLEMENTARY TABLE 2: REAGENTS 3
SUPPLEMENTARY TABLE 3: ANTIBODIES 4
SUPPLEMENTARY TABLE 4: TAQMAN® PROBES 6

SUPPLEMENTARY FIGURES **7**

SUPPLEMENTARY FIGURE 1. ELIGLUSTAT INCREASES TRABECULAR BONE IN HEALTHY MALE MICE BY INHIBITING OC *IN VIVO* 9
SUPPLEMENTARY FIGURE 2. ELIGLUSTAT AMELIORATES 5TGM1-GFP MM CELL-INDUCED BONE DISEASE IN MALE MICE BUT HAS NO EFFECT ON OC PROGENITORS OR MM TUMOUR BURDEN 11
SUPPLEMENTARY FIGURE 3. ELIGLUSTAT DID NOT ALTER OB AND CORTICAL BONE IN HFD INDUCED MGUS CONDITION 13
SUPPLEMENTARY FIGURE 4. ELIGLUSTAT COMBINED WITH ZA DOES NOT ENHANCE OB NUMBER OR AREA 15
SUPPLEMENTARY FIGURE 5. ELIGLUSTAT INHIBITS OC FORMATION VIA TRAF3 17
SUPPLEMENTARY FIGURE 6. ELIGLUSTAT ACCUMULATES LAMP2 AND TRAF3 IN A CELL, IN ADDITION, AUTOPHAGY INHIBITORS INCLUDING SAR405, 3MA, BAFA1 AND CQ PREVENT OC FORMATION 20
SUPPLEMENTARY FIGURE 7. WESTERN BLOT FULL SCANS 21

SUPPLEMENTARY TABLE

Supplementary Table 1: Baseline MM patient characteristics

**Patient Characteristics** (n=9)	
**Sex**	M 8 (89%)<br>F 1 (11%)
**Median Age**	61 (41-62)
**ISS**<br>**Stage 1**<br>**Stage 2**<br>**Stage 3**<br>**Unknown**	 1 (11%)<br>4 (44%)<br>1 (11%)<br>3 (34%)
**Cytogenetics**<br>**Standard Risk**<br>**High Risk**<br>**Unknown**	 4 (44%)<br>3 (34%)<br>2 (22%)
**Disease response:**<br>**CR/VGPR**<br>**PR/MR**<br>**PD**	 5 (56%)<br>2 (22%)<br>2 (22%)
**Median lines of chemotherapy**<br>**ASCT**	2 (22%)<br>7 (78%)
**Bony disease at time of diagnosis**<br>**Spinal disease**<br>**Pelvic disease**	8 (89%)<br>7 (78%)<br>1 (11%)
**Bisphosphonate therapy (zoledronic acid)**	8 (89%)
Abbreviations: ISS Stage 1: B2 microglobulin < 3.5mg/L and Albumin>35g/L, ISS Stage 3: B2 microglobulin>5.5mg/L, ISS stage 2: patients not fulfilling criteria for Stage 1 or 3. Adverse cytogenetics defined as per IMWG criteria: [(t(4;14), t(14:16), t(14;20) 1q gain or del 17p]. Disease response defined as per IMWG criteria CR: complete response, VGPR: very good partial response, PR: partial response, MR: minimal response, PD: progressive disease. ASCT: autologous stem cell transplantation	

Supplementary Table 2: Reagents

Reagent	Source	Catalog number	Note
RPMI-1640	Sigma-Aldrich	R8758-500ML	
Fetal Bovine Serum	Gibco	10500064	
	Sigma-Aldrich	F2442	
Penicillin-Streptomycin	Sigma-Aldrich	P0781	1:100 dilution
L-Glutamine	Sigma-Aldrich	G7513-100ML	1:100 dilution
Minimum Essential Medium Non-Essential Amino Acids	Gibco	11140050	1:100 dilution
Sodium Pyruvate	Gibco	11360-070	1:100 dilution
Dulbecco's Modified Eagle Medium (DMEM)	Sigma-Aldrich	D6429-500ML	
Alamar blue Cell Viability Reagent	Thermo Fisher	DAL1025	
RBC Lysis Buffer	Sigma	R7757-100ML	
NR12S dye	Bio-Techne Ltd.	7509	
Eliglustat	Selleckchem	S7852	stored at -20°C until diluted in DMSO immediately before use in culture medium (0.1–50 μM)
	provided by Genzyme, a Sanofi Company	NA	
Murine sRANK Ligand (RANKL)	peprotech	315-11C-10	
Mouse M-CSF	Miltenyi Biotec	130-101-704	
Recombinant Human sRANK Ligand (E.coli derived)	peprotech	310-01	
Recombinant Human M-CSF Protein	R&D Systems	216-MC	
Minimum Essential Medium (MEM) Alpha	Gibco	12571-063	
Zoledronic acid	Sigma	1724827-150MG	
Iodomethane-d_3	Merck	176036-5G	
C16 Glucosyl(ß) Ceramide (d18:1/16:0); C16GlcCer	Avanti Polar Lipids	860539P-5MG	dissolved in 100% methanol at 37°C and then stored at -20°C until use for cell culture
C16 Lactosyl(ß) Ceramide (d18:1/16:0); C16LacCer	Avanti Polar Lipids	860576P-5MG	
C24 Lactosyl(ß) Ceramide (d18:1/24:0); C24LacCer	Avanti Polar Lipids	860577P-5MG	
TRAP kit	Sigma-Aldrich	386A	
Methyl Green	Bioenno Lifesciences	003027	
Osmium Tetroxide	Honeywell Fulka	251755-2ml	
D-*threo*-PDMP (D-PDMP)	Matreya LLC	1756	
	Santa Cruz	sc-280659	

Mouse IgG2bκ ELISA Quantification Set	Bethyl Laboratories, Inc	E90-109	
Immunofluorescence staining mounting medium	FluorSave™ Reagent	345789-20ML	
Lysosome Isolation Kit	Sigma-Aldrich	LYSISO1	

Supplementary Table 3: Antibodies

**Reagent**	**Source**	**Clone**	**Catalog number**	**Dilution**	**Validation**
Antibodies used for flow cytometry					
CD16/CD32 Monoclonal Antibody (FcR Block)	Thermo Fisher	93	14-0161-85	200	C57BL/KaLwRijHsd and CD45.1+ B6.SJL splenocytes and bone marrow cells
Zombie Aqua™ Fixable Viability Kit	BioLegend	NA	423102	400	C57BL/KaLwRijHsd and CD45.1+ B6.SJL splenocytes and bone marrow cells
Brilliant Violet 605™ anti-mouse/human CD11b Antibody	BioLegend	M1/70	101237	200	C57BL/KaLwRijHsd bone marrow cells
APC anti-mouse CD115 (CSF-1R) Antibody	BioLegend	AFS98	135510	200	C57BL/KaLwRijHsd bone marrow cells
PE anti-mouse CD117 (c-Kit) Antibody	BioLegend	2B8	105808	200	C57BL/KaLwRijHsd bone marrow cells
Alexa Fluor® 700 anti-mouse/human CD45R/B220 Antibody	BioLegend	RA3-6B2	103232	200	C57BL/KaLwRijHsd bone marrow cells
Brilliant Violet 421™ anti-mouse CD3 Antibody	BioLegend	17A2	100227	200	C57BL/KaLwRijHsd bone marrow cells
Pacific Blue™ anti-mouse	BioLegend	A20	110722	100	CD45.1+ B6.SJL bone

CD45.1 Antibody					marrow cells
Alexa Fluor® 700 anti-mouse CD45.2 Antibody	BioLegend	104	109821	100	$CD45.1^+$ B6.SJL bone marrow cells
CD11b Monoclonal Antibody, APC	eBioscience	M1/70	17-0112-82	200	$CD45.1^+$ B6.SJL bone marrow cells
CD11c Monoclonal Antibody, FITC	eBioscience	N418	11-0114-82	200	$CD45.1^+$ B6.SJL bone marrow cells
Antibodies used for Western blot					
LC3	Sigma-Aldrich	Polyclonal	L8918	1000	RAW264.7 cell
TRAF3	Cell Signaling	Polyclonal	4729	1000	C57BL/6J bone marrow cells and RAW264.7 cell
P62 (SQSTM1)	MBL	Polyclonal	PM045	1000	RAW264.7 cell
TRAF6	abcam	Monoclonal	ab33915	1000	C57BL/6J bone marrow cells
IkBα	Cell Signaling	44D4	4812S	1000	C57BL/6J bone marrow cells
LAMP1	abcam	Polyclonal	ab24170	1000	RAW264.7 cell
βActin	Cell Signaling	8H10H10	3700	5000	C57BL/6J bone marrow cells and RAW264.7 cell
IRDye 800CW Donkey Anti-Rabbit IgG Secondary Antibody	LI-COR		926-32213 RRID AB_621848	10,000	validated by manufacturer
IRDye 680LT Donkey Anti-Mouse IgG Secondary Antibody	LI-COR		926-68022 RRID AB_10715072	20,000	validated by manufacturer
Antibodies used for confocal microscopy					
LAMP2	Santa Cruz Biotechnology	H4B4	sc18822	200 (Sup. Fig. 6a)	U2OS cell
Anti-LAMP2 antibody	abcam	GL2A7	ab13524	200 (Figure	RAW264.7 cell

				6o and Sup. Fig. 6c)	
Cy3-AffiniPure Goat Anti-Mouse IgG (H+L), Secondary	Jackson ImmunoResearch	Polyclonal	115-165-003	500 (Sup. Fig. 6a)	U2OS cell
CoraLite®488-conjugated TRAF3 Monoclonal antibody	Proteintech	1E3F4	CL488-66310	150 (Figure 6o and Sup. Fig. 6c)	RAW264.7 cell
Alexa Fluor™ 647 chicken anti-rat IgG (H+L)	Invitrogen™	Polyclonal	A21472	500 (Figure 6o)	RAW264.7 cell
DAPI	Thermo Scientific		62248	2μg/ml	U2OS cell and RAW264.7 cell

Supplementary Table 4: TaqMan® probes

Gene	Source	Identifier
traf3	Thermo Fisher	Mm00495752_m1
gapdh	Thermo Fisher	Mm99999915_g1

SUPPLEMENTARY FIGURES

Supplemental Figure 1

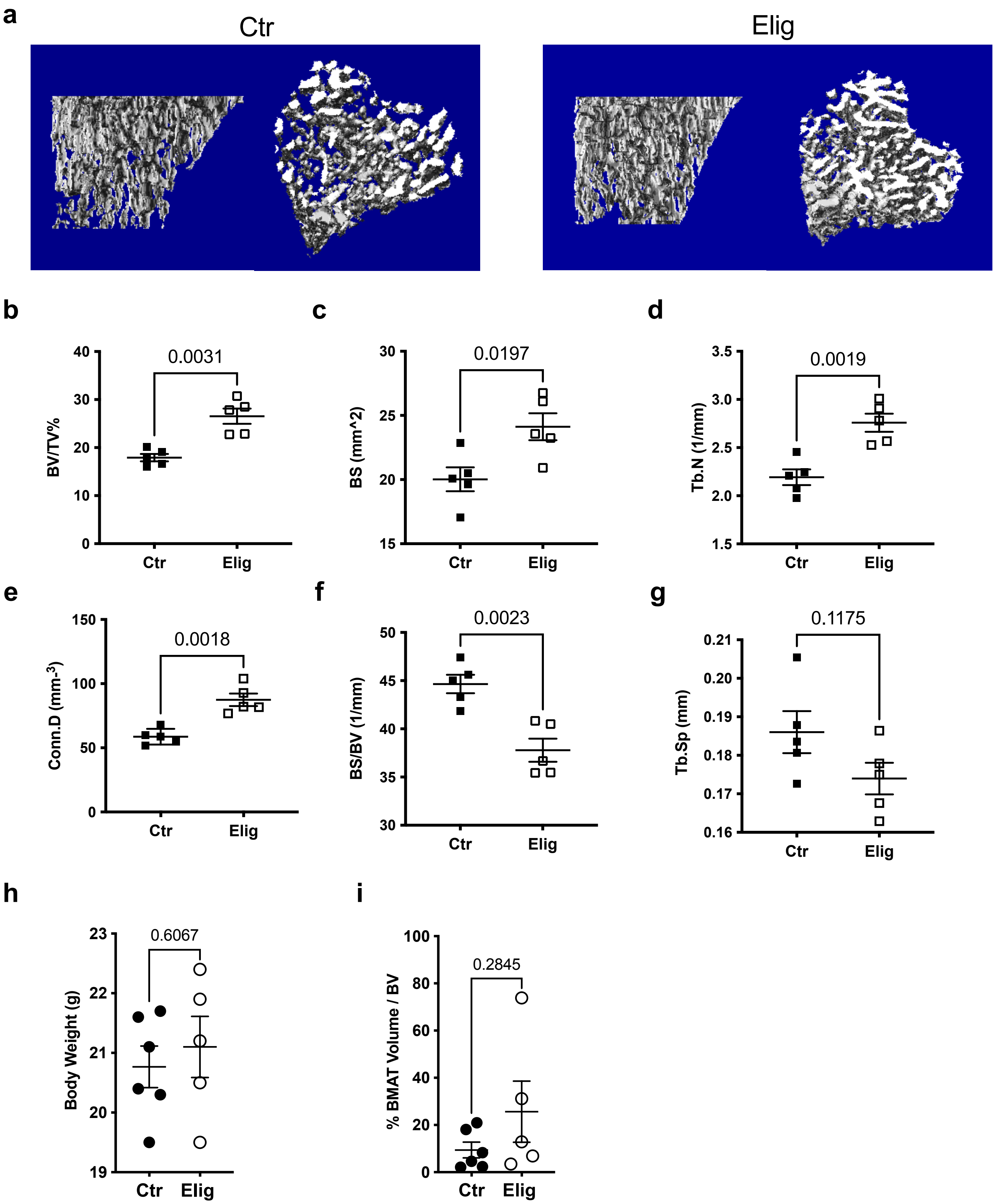

Supplementary Figure 1. Eliglustat increases trabecular bone in healthy male mice by inhibiting OC *in vivo*.

(**a**) Representative micro-CT reconstruction images of tibiae from 8-week-old male C57BL/6J mice treated with normal chow (Ctr, n=5 biologically independent animals) or eliglustat (Elig, n=5 biologically independent animals) chow for 19 days. (**b-g**) Micro-CT analysis of tibiae: BV/TV, BS, Tb.N, Conn.D, BS/BV and Tb.Sp (Ctr, n=5 and Elig, n=5 biologically independent animals). (**h, i**) The body weight (**h**) and BMAT volume (**i**) of the mice from respective group (Ctr, n=5 and Elig, n=5 biologically independent animals). Data are presented as mean values +/- SEM. Exact *p* values are depicted in the figure. Statistical analysis was performed using unpaired two-tailed Student's *t* test. Source data are provided as a Source Data file.

Supplemental Figure 2

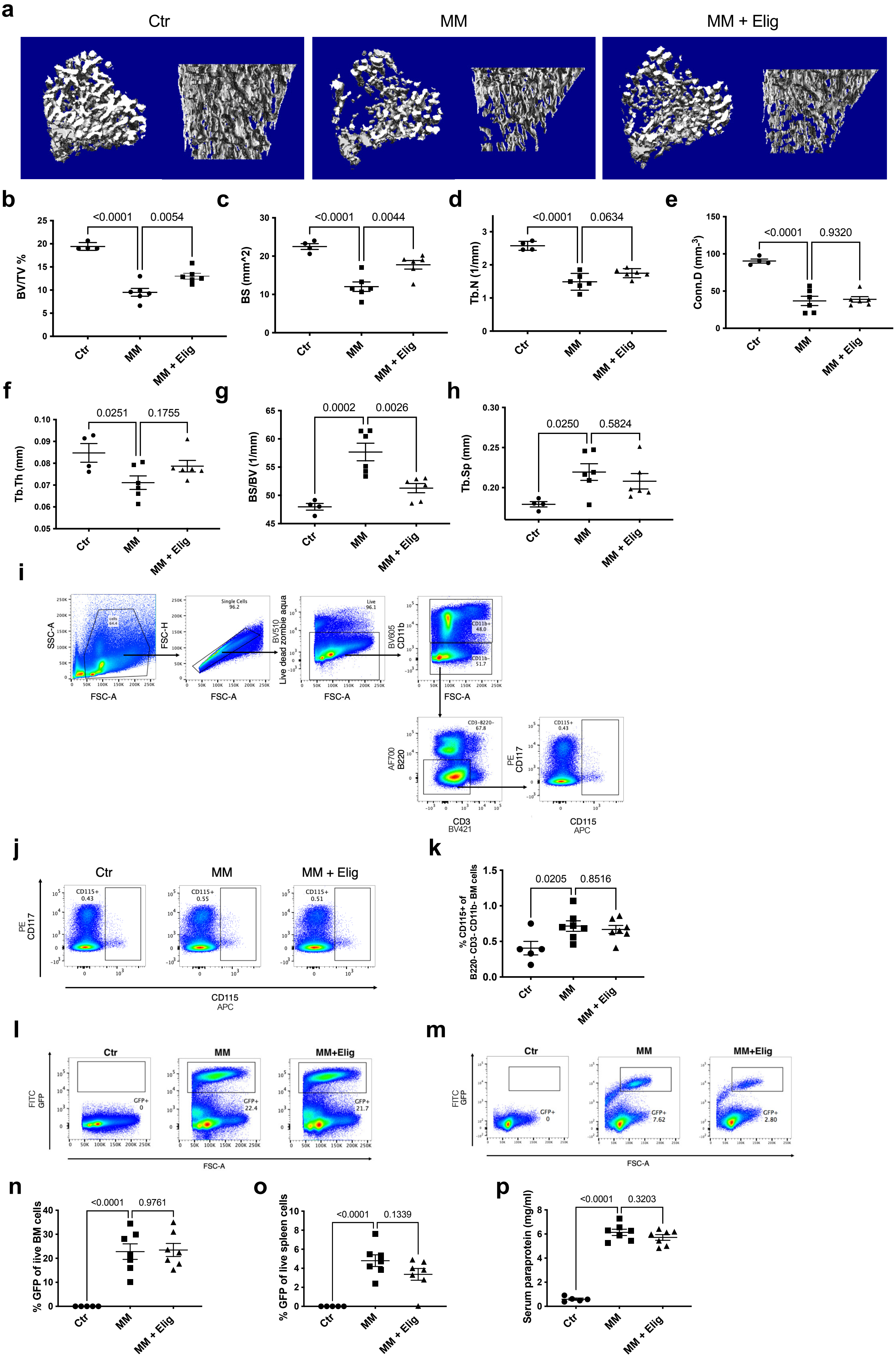

Supplementary Figure 2. Eliglustat ameliorates 5TGM1-GFP MM cell-induced bone disease in male mice but has no effect on OC progenitors or MM tumour burden.

5TGM1-GFP MM cells were injected to 8-week-old male C57BL/KaLwRijHsd mice to generate the MM model. Eliglustat chow (150 mg/kg/day) was administered from day 4 post tumour injection (until day 23). (**a**) Representative micro-CT reconstruction images of tibiae trabecular bones from naive control (Ctr, n=4 biologically independent animals), MM mice with normal chow (MM, n=6 biologically independent animals), or MM mice with eliglustat chow (MM+Elig, n=6 biologically independent animals). (**b-h**) Tibiae trabecular bone parameters were assessed: BV/TV, BS, Tb.N, Conn.D, Tb.Th, BS/BV and Tb.Sp (Ctr, *n*=4; MM, *n*=6 and MM+Elig, *n*=6 biologically independent animals).

(**i-k**) Flow cytometry gating strategy of the OC progenitors (CD11b- B220- CD3- CD115+) in BM cells (**i**) from female C57BL/KaLwRijHsd mice, either untreated controls (Ctr), MM mice with normal chow (MM), or MM mice with eliglustat chow (MM+Elig). Representative plots of OC progenitors (CD11b- B220- CD3- CD115+) from the indicated female groups (**j**) and the quantified percentage (**k**) (Ctr, *n*=5; MM, *n*=7 and MM+Elig, *n*=7 biologically independent animals). (**l-o**) Female mice tumour burden in BM (**l**) and spleen (**m**) was assessed by quantifying GFP+ cells using flow cytometry (**n-o**) (Ctr, *n*=5; MM, *n*=7 and MM+Elig, *n*=7 biologically independent animals). (**p**) Serum paraprotein IgG2b*κ* secreted by 5TGM1-GFP cells was quantified using ELISA (Ctr, *n*=5; MM, *n*=7 and MM+Elig, *n*=7 biologically independent animals).

Data are presented as mean values +/- SEM. Exact *p* values are depicted in the figure. Statistical analysis was performed using One-way ANOVA. Source data are provided as a Source Data file.

Supplemental Figure 3

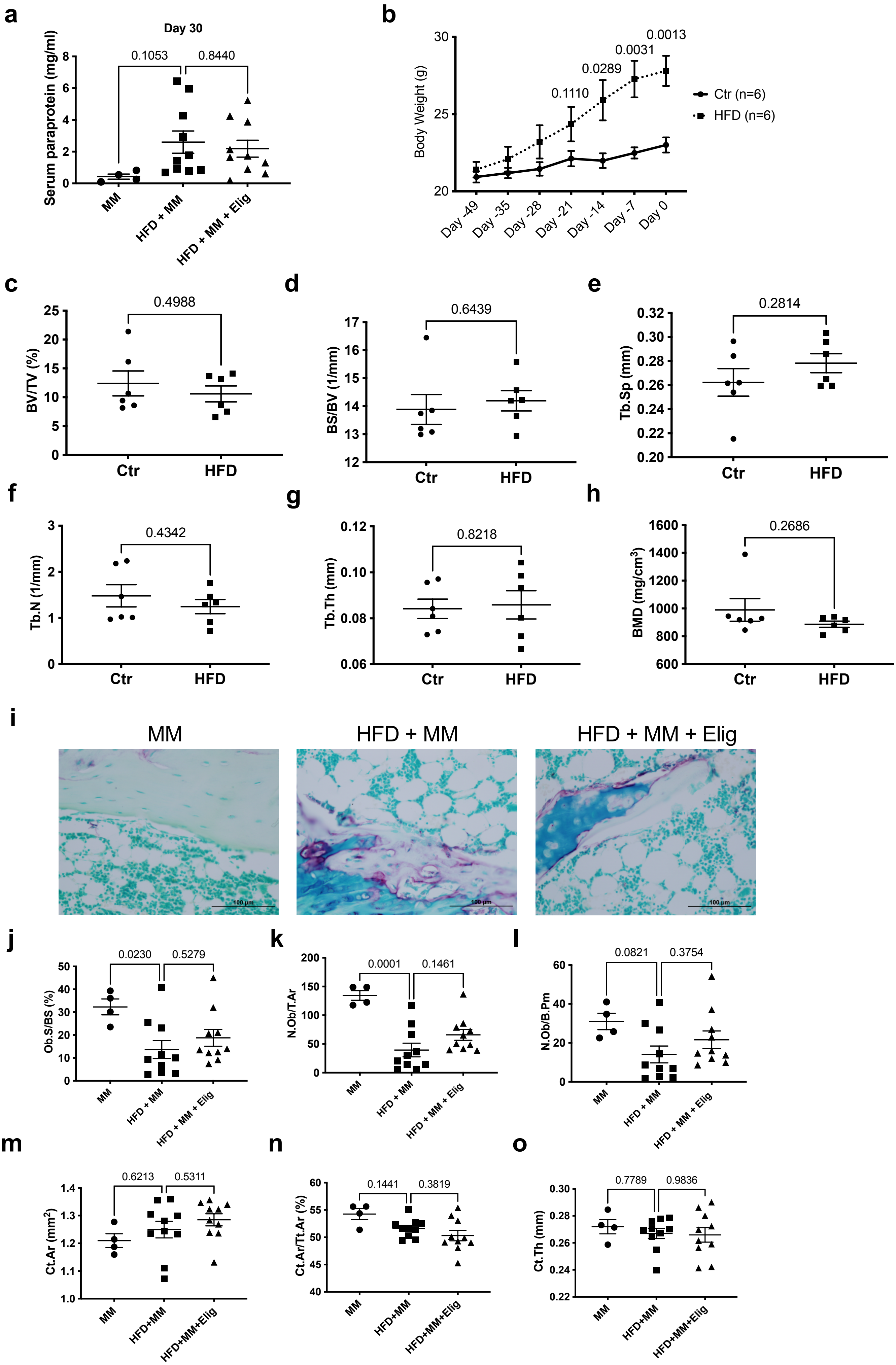

Supplementary Figure 3. Eliglustat did not alter OB and cortical bone in HFD induced MGUS condition.

(**a**) Eliglustat does not alter serum paraprotein levels in MM, HFD+MM and HFD+MM+Elig groups measured at day 30 post MM cell injection (MM, *n*=4; HFD+MM, *n*=10 and HFD+MM+Elig, *n*=10 biologically independent animals). Statistical analysis was performed using One-way ANOVA for **a**.

(**b**) C57BL6J mice were divided into normal diet group (Ctr, n=6 biologically independent animals) or HFD group (HFD, n=6 biologically independent animals), body weight changes were observed over 49 days. (**c-h**) Micro-CT analysis parameters of tibiae: BV/TV, BS/BV, Tb.Sp, Tb.N, Tb.Th and BMD for mice on Ctr diet and HFD (Ctr, *n*=6 and HFD, *n*=6 biologically independent animals). Statistical analysis was performed using unpaired two-tailed Student's *t* test for **b-h**.

(**i-l**) Histology images and bone histomorphometry analysis for OB including Ob.S/BS, N.Ob/T.Ar and N.Ob/B.Pm in MM, HFD+MM and HFD+MM+Elig groups (MM, *n*=4; HFD+MM, *n*=10 and HFD+MM+Elig, *n*=10 biologically independent animals). The result is representative of two independent experiments. (**m-o**) Cortical bone parameters including Ct.Ar (cortical area), Ct.Ar/Tt.Ar (cortical area over total area) and Ct.Th (cortical thickness) for MM, HFD+MM, HFD+MM+Elig groups (MM, *n*=4; HFD+MM, *n*=10 and HFD+MM+Elig, *n*=10 biologically independent animals). Statistical analysis was performed using One-way ANOVA for **j-o**.

Data are presented as mean values +/- SEM. Exact *p* values are depicted in the figure. Source data are provided as a Source Data file.

Supplemental Figure 4

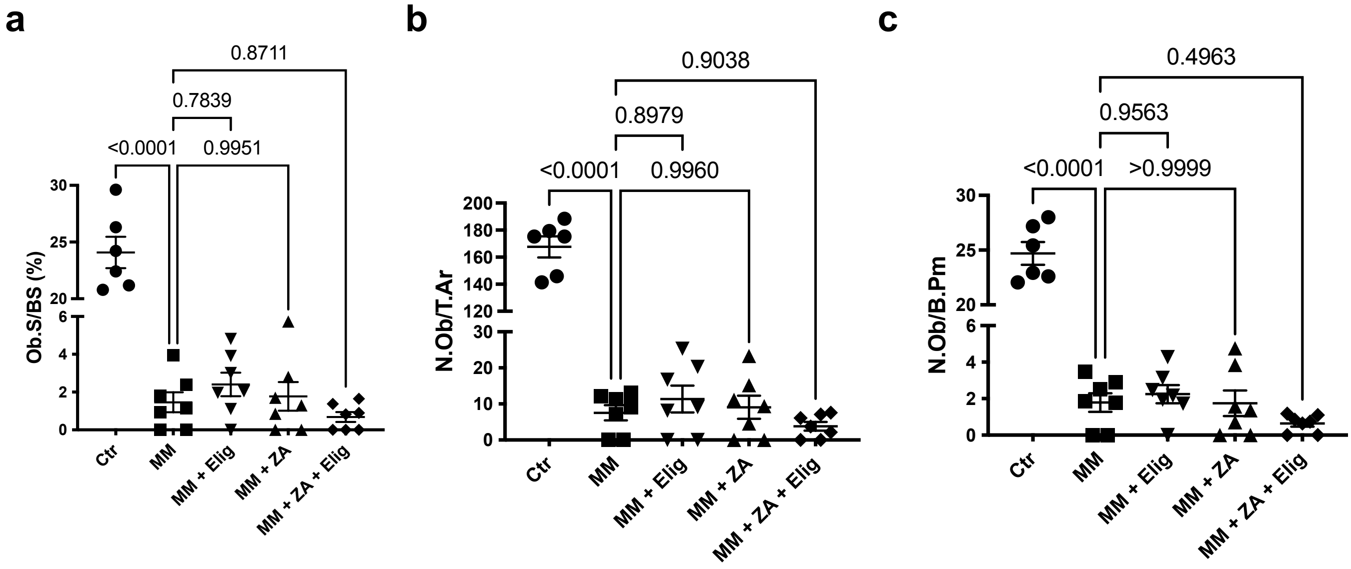

Supplementary Figure 4. Eliglustat combined with ZA does not enhance OB number or area. (**a-c**) Bone histomorphometric analysis for OB parameters including Ob.S/BS, N.Ob/T.Ar and N.Ob/B.Pm were based on morphology in paraffin sections stained with TRAP and methyl green and quantified using *Osteomeasure software* (Ctr, *n*=6; MM, *n*=7; MM+Elig, *n*=7; MM+ZA, *n*=7; MM+ZA+Elig, *n*=7 biologically independent animals). Data are presented as mean values +/- SEM. Statistical analysis was performed using One-way ANOVA. Exact *p* values are depicted in the figure. Source data are provided as a Source Data file.

Supplemental Figure 5

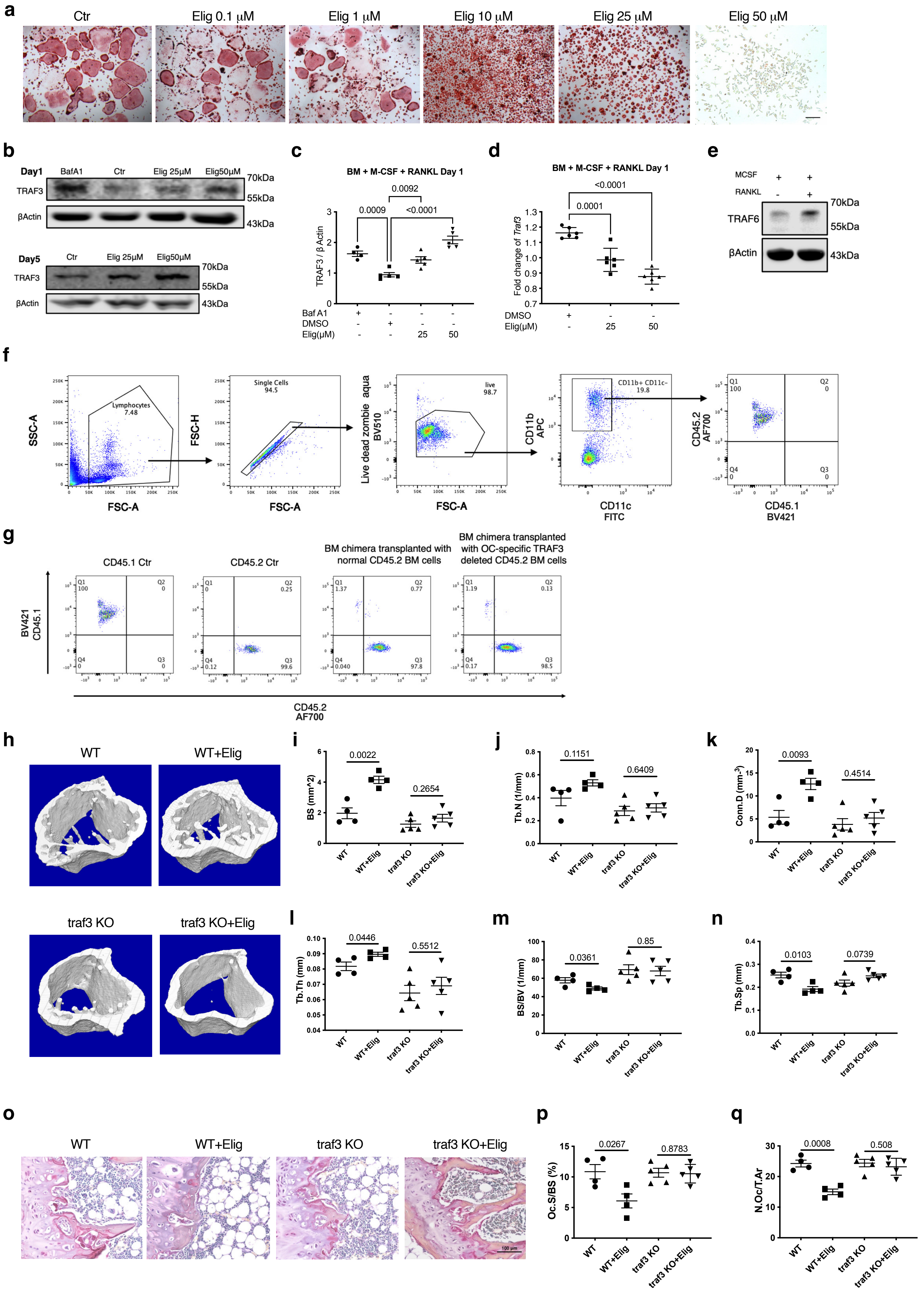

Supplementary Figure 5. Eliglustat inhibits OC formation via TRAF3.

(**a**) 8-week-old C57BL/6J mouse BM cells were treated with M-CSF and RANKL to form OCs in 96-well plates. Different doses of Eliglustat (0.1, 1, 10, 25, 50 μM) were added and OCs were identified by TRAP staining on day 6. Scale bar represents 200μm.

(**b-c**) TRAF3 protein levels were quantified by Western blot on day 1 of OC differentiation (similar pattern was observed throughout the differentiation period including on day 5). BafA1 (10nM) was added 2 hours before protein harvest; BafA1, n=4; DMSO control, n=5; 25μM Elig, n=5; 50μM Elig, n=5 biologically independent samples. The result is representative of four independent experiments. Statistical analysis was performed using One-way ANOVA for **c**.

(**d**) *traf3* mRNA levels on day 1 were quantified by qRT-PCR; DMSO control, n=6; 25μM Elig, n=6; 50μM Elig, n=6 biologically independent samples. Statistical analysis was performed using One-way ANOVA for **d**.

(**e**) 50ng/ml RANKL increased TRAF6 protein level in primary BM cells during OC formation after 2 hours treatment. The result is representative of three independent experiments.

(**f, g**) CD45.1$^+$ recipient mice were lethally irradiated and reconstituted with CD45.2$^+$ wildtype BM cells or CD45.2$^+$ OC-specific TRAF3-deleted BM cells. Gating strategy for checking the reconstitution efficacy of the chimeric mice by evaluating the peripheral blood CD45.2$^+$ myeloid population (**f**). Representative flow cytometry plots showing the distribution of CD45.1$^+$/CD45.2$^+$ of myeloid cells in peripheral blood from an unirradiated CD45.1 mouse, unirradiated CD45.2 mouse, irradiated CD45.1$^+$ mouse transplanted with wildtype CD45.2$^+$ BM cells and CD45.1$^+$ mouse transplanted with CD45.2$^+$ OC-specific TRAF3-deleted BM cells (**g**). (**h**) Micro-CT reconstruction images of WT, WT+Elig, traf3 KO and traf3 KO+Elig mice. (**i-n**) Micro-CT analysis parameters of tibiae including: BS, Tb.N, Conn.D, Tb.Th, BS/BV and Tb.Sp (WT, *n*=4; WT+Elig, *n*=4; traf3 KO, *n*=5; traf3 KO + Elig, *n*=5 biologically independent animals). (**o**) Representative TRAP/0.2% methyl green stained tibial sections showing red OCs on the

endocortical bone surface from each group. The result is representative of two independent experiments. (**p, q**) Bone histomorphometry parameters including Oc.S/BS and N.Oc/T.Ar (WT, *n*=4; WT+Elig, *n*=4; traf3 KO, *n*=5; traf3 KO + Elig, *n*=5 biologically independent animals). Statistical analysis was performed using unpaired two-tailed Student's *t* test for **i-n** and **p, q**. Data are presented as mean values +/- SEM. Exact *p* values are depicted in the figure. Source data are provided as a Source Data file.

Supplemental Figure 6

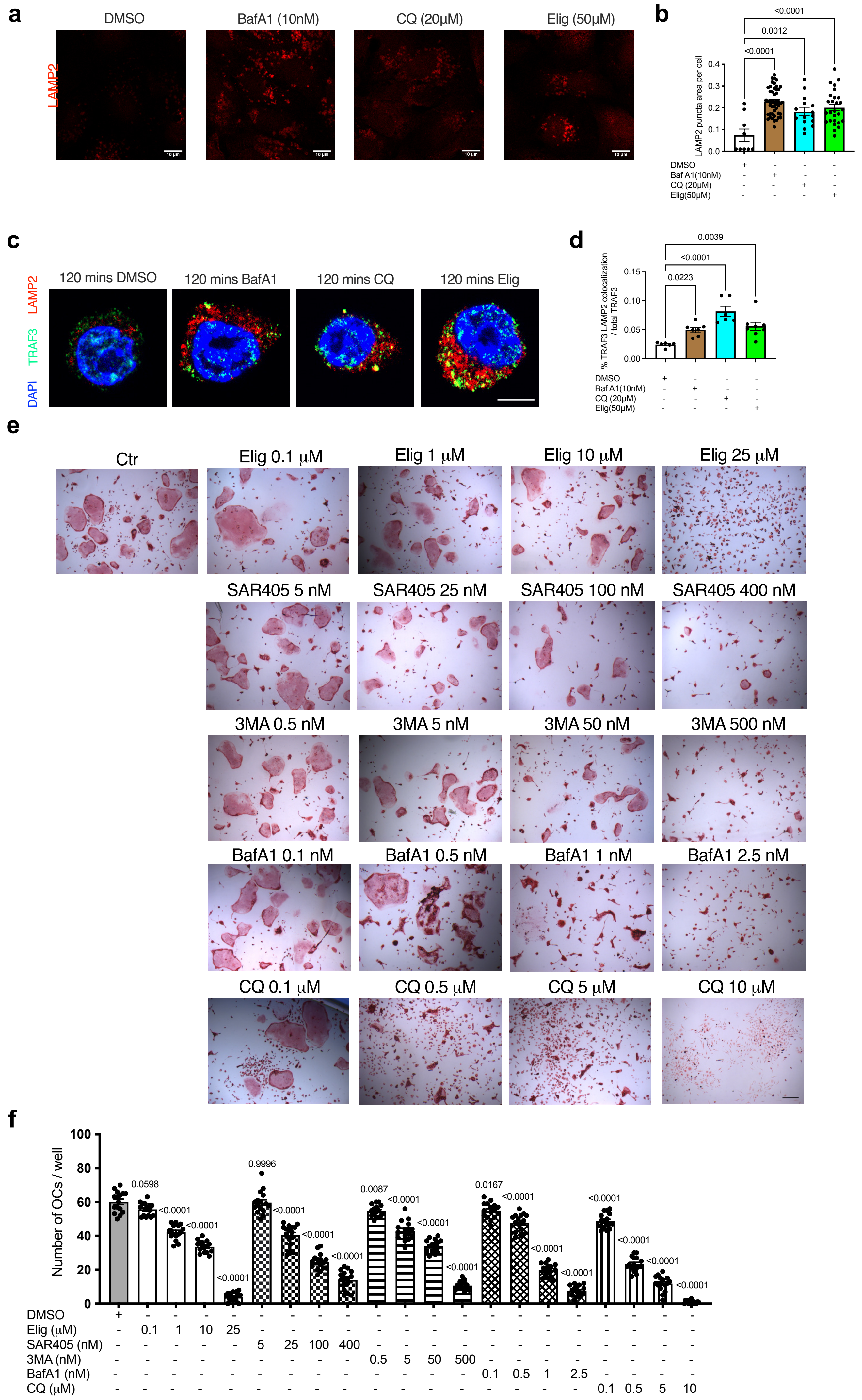

Supplementary Figure 6. Eliglustat accumulates LAMP2 and TRAF3 in a cell, in addition, autophagy inhibitors including SAR405, 3MA, BafA1 and CQ prevent OC formation.

(**a, b**) Confocal images (**a**) and quantification (**b**) of the LAMP2 puncta area per eliglustat-treated U2OS cell (arbitrary units). CQ and BafA1 served as the positive control. n=9 cells for DMSO control, n=47 cells for BafA1, n=15 cells for CQ and n=27 cells for Elig group. The result is representative of three independent experiments.

(**c, d**) Confocal images of TRAF3 and LAMP2 in Pre-OCs derived from RAW264.7 cells were treated with BafA1, CQ and eliglustat for 120 minutes, Scale bar represents 10μm, n≥4 (**c**); the percentage of TRAF3 and LAMP2 colocalization is quantified (**d**). n=6 cells for DMSO control, n=7 cells for BafA1, n=6 cells for CQ and n=8 cells for Elig group. The result is representative of three independent experiments.

(**e**) BM cells were differentiated into OCs with M-CSF and RANKL. Different doses of eliglustat (0.1, 1, 10, 25 μM), SAR405 (5, 25, 100, 400 nM), 3MA (0.5, 5, 50, 500 nM), BafA1 (0.1, 0.5, 1, 2.5 nM) and CQ (0.1, 0.5, 5, 10 μM) were present throughout the culture period and OCs were identified by TRAP staining on day 5. Scale bar represents 200μm. (**f**) Number of OCs per well was quantified. *n*=16 biologically independent samples examined over 4 independent experiments.

Data are presented as mean values +/- SEM. Exact *p* values are depicted in the figure. Statistical analysis was performed using One-way ANOVA. Source data are provided as a Source Data file.

Supplementary Figure 7. Western blot full scans.

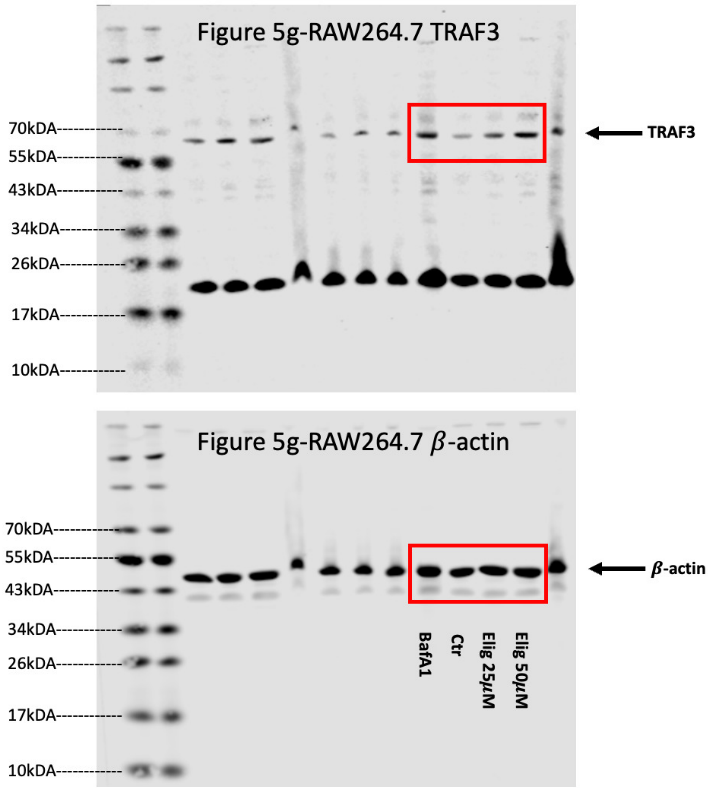

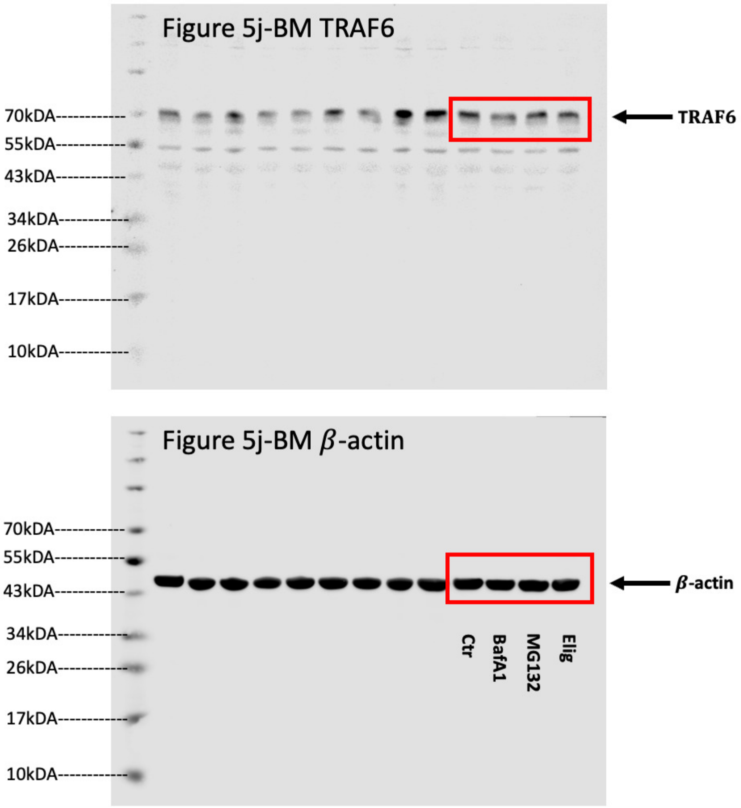

Supplementary Figure 7. Western blot full scans (continued).

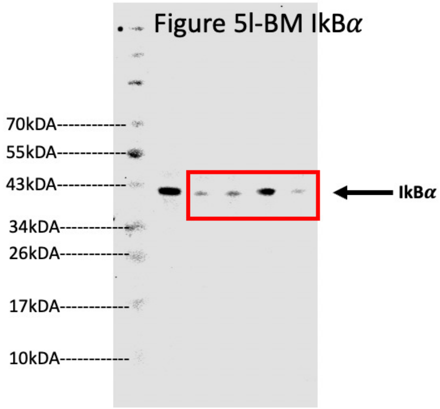

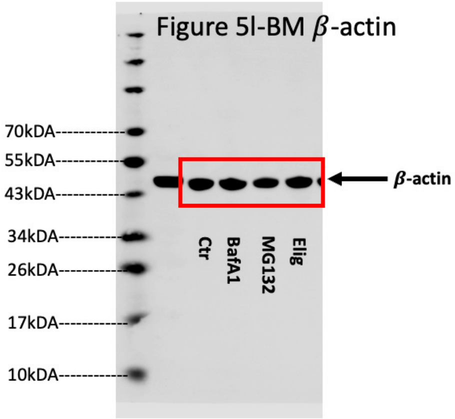

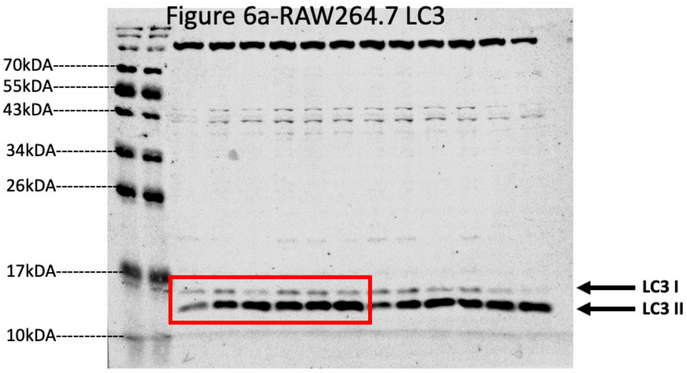

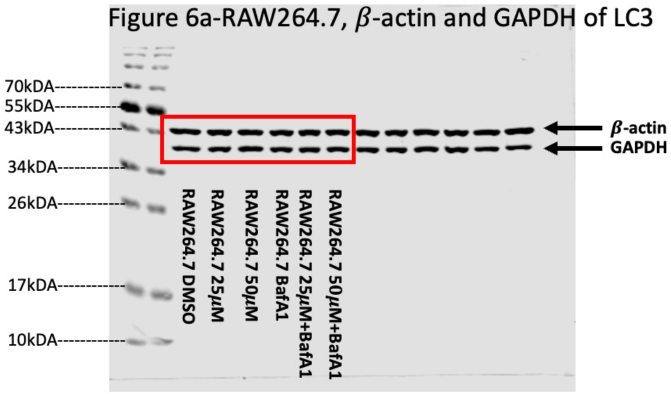

Supplementary Figure 7. Western blot full scans (continued).

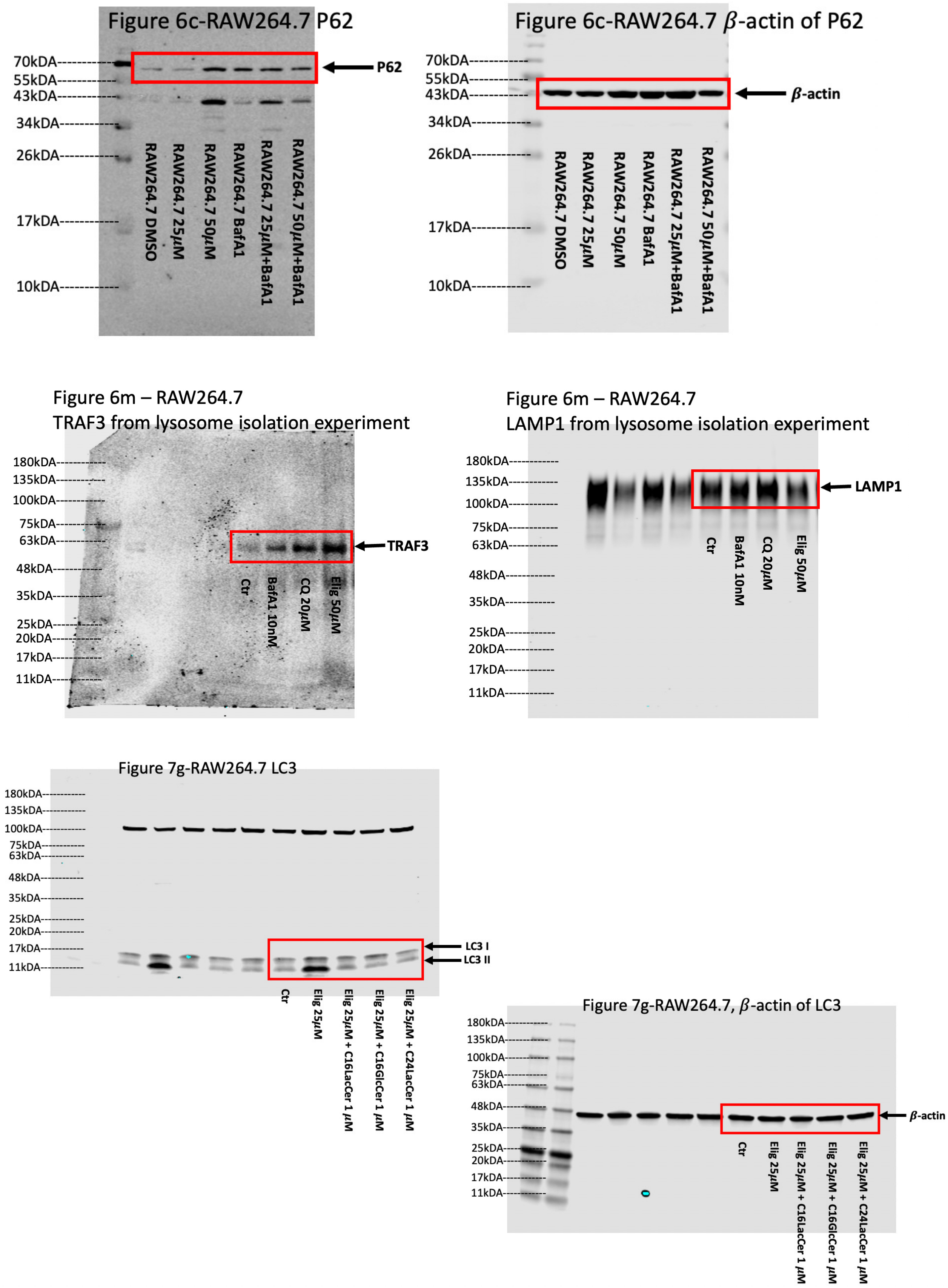

Supplementary Figure 7. Western blot full scans (continued).

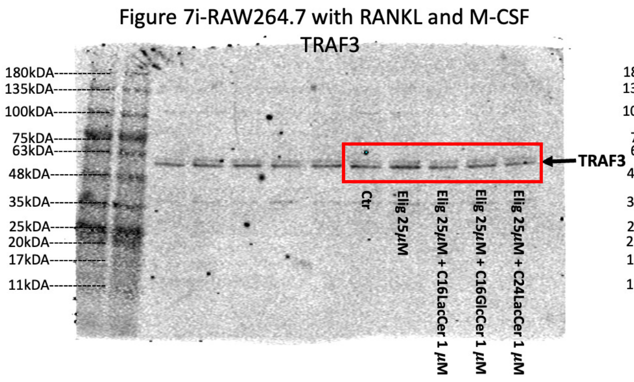

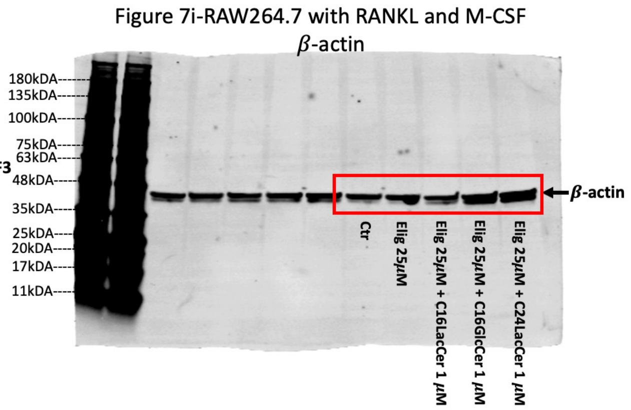

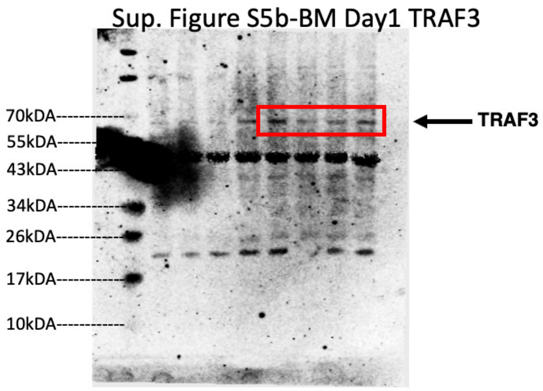

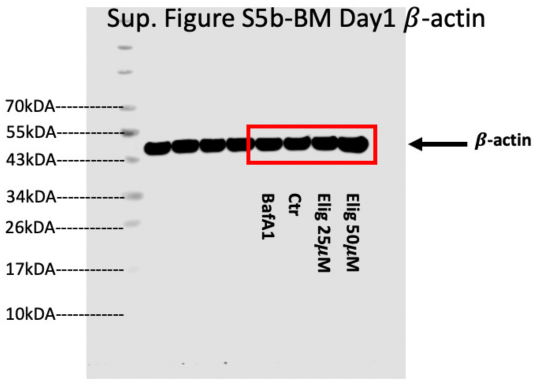

Supplementary Figure 7. Western blot full scans (continued).

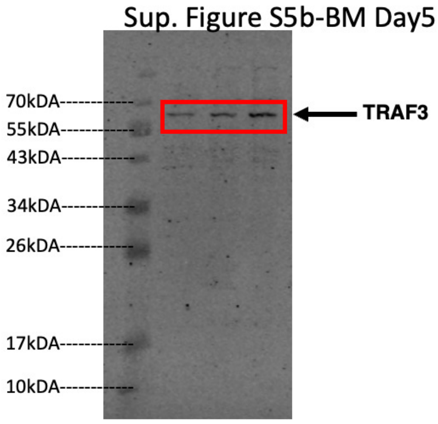

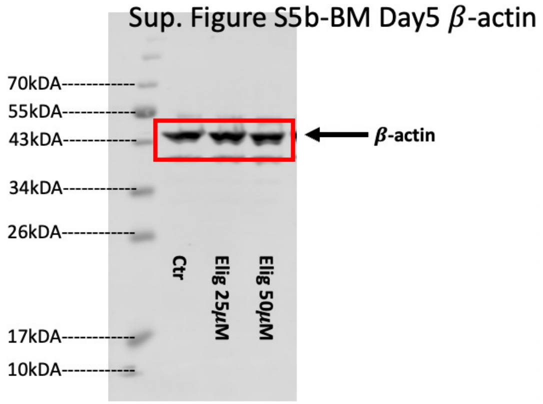

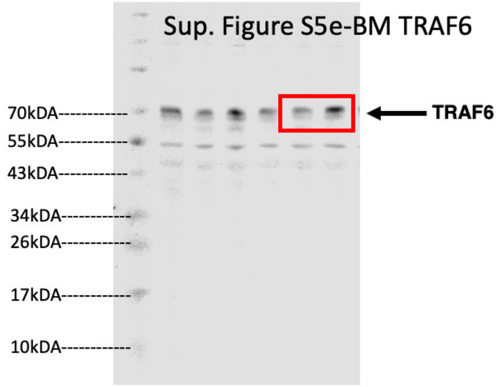

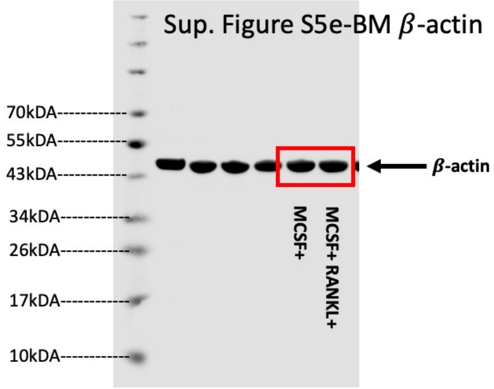

Supplement: Supplementary file 1 — Supplementary Information [file 41467_2022_35358_MOESM1_ESM.pdf]
