# Supplementary material for: Modulating glycosphingolipid metabolism and autophagy improves outcomes in pre-clinical models of myeloma bone disease
Source: Nat Commun. 2022 Dec 22;13:7868. doi: 10.1038/s41467-022-35358-3 (PMC9780346; doi:10.1038/s41467-022-35358-3)
Supplement: Supplementary file 3 — Description of Additional Supplementary File [file 41467_2022_35358_MOESM3_ESM.docx]

**Description of Additional Supplementary Files**

**Supplementary Data 1.** Original full lipidomics data.
