# Supplementary material for: Modulating glycosphingolipid metabolism and autophagy improves outcomes in pre-clinical models of myeloma bone disease
Source: Nat Commun. 2022 Dec 22;13:7868. doi: 10.1038/s41467-022-35358-3 (PMC9780346; doi:10.1038/s41467-022-35358-3)
Supplement: Supplementary file 5 — Reporting Summary [file 41467_2022_35358_MOESM5_ESM.pdf]

nature portfolio

Corresponding author(s): Nicole J. Horwood
Anna Katharina Simon

Last updated by author(s): Oct 9, 2022

Reporting Summary

Nature Portfolio wishes to improve the reproducibility of the work that we publish. This form provides structure for consistency and transparency in reporting. For further information on Nature Portfolio policies, see our Editorial Policies and the Editorial Policy Checklist.

Statistics

For all statistical analyses, confirm that the following items are present in the figure legend, table legend, main text, or Methods section.

n/a	Confirmed	
☐	☒	The exact sample size (*n*) for each experimental group/condition, given as a discrete number and unit of measurement
☐	☒	A statement on whether measurements were taken from distinct samples or whether the same sample was measured repeatedly
☐	☒	The statistical test(s) used AND whether they are one- or two-sided<br>*Only common tests should be described solely by name; describe more complex techniques in the Methods section.*
☒	☐	A description of all covariates tested
☒	☐	A description of any assumptions or corrections, such as tests of normality and adjustment for multiple comparisons
☐	☒	A full description of the statistical parameters including central tendency (e.g. means) or other basic estimates (e.g. regression coefficient) AND variation (e.g. standard deviation) or associated estimates of uncertainty (e.g. confidence intervals)
☐	☒	For null hypothesis testing, the test statistic (e.g. *F*, *t*, *r*) with confidence intervals, effect sizes, degrees of freedom and *P* value noted<br>*Give P values as exact values whenever suitable.*
☒	☐	For Bayesian analysis, information on the choice of priors and Markov chain Monte Carlo settings
☒	☐	For hierarchical and complex designs, identification of the appropriate level for tests and full reporting of outcomes
☒	☐	Estimates of effect sizes (e.g. Cohen's *d*, Pearson's *r*), indicating how they were calculated

Our web collection on statistics for biologists contains articles on many of the points above.

Software and code

Policy information about availability of computer code

Data collection	The ViiA 7 Real-Time PCR System was used to conduct the quantitative PCR.<br>Odyssey CLx Imaging System is used to collect the Western Blot data.<br>Tibiae were scanned with the PerkinElmer QuantumFX scanner.<br>The Olympus BX51 microscope or Hamamatsu NanoZoomer S210 equipped with osteomeasure (Osteometrics) software were used to quantify OC parameters.<br>Zeiss LSM 980 Airyscan confocal system,Olympus Fluoview FV3000, Leica TCS SP8, Zeiss LSM 780 microscope.<br>Transmission electron microscopy HITACHI 7650<br>Linear ion-trap mass spectrometer (LTQ-XL, Thermo Finnigan, USA)
Data analysis	FlowJo 10.2, GraphPad Prism9 (San Diego, CA)., Image Studio Lite, Analyse-12.0 software, ImageJ 2.1.0/1.53c (National Institutes of Health, USA); arivis Vision4D X64 software and MATLAB version 9.9 (R2020b)

For manuscripts utilizing custom algorithms or software that are central to the research but not yet described in published literature, software must be made available to editors and reviewers. We strongly encourage code deposition in a community repository (e.g. GitHub). See the Nature Portfolio guidelines for submitting code & software for further information.

Data

Policy information about availability of data

All manuscripts must include a data availability statement. This statement should provide the following information, where applicable:

- Accession codes, unique identifiers, or web links for publicly available datasets
- A description of any restrictions on data availability
- For clinical datasets or third party data, please ensure that the statement adheres to our policy

The data supporting the findings from this study are available within the manuscript and the supplementary information.
Accession codes, unique identifiers, or web links for publicly available datasets -- N/A
A description of any restrictions on data availability -- N/A
For clinical datasets or third party data, please ensure that the statement adheres to our policy -- We provided the clinical dataset in Supplementary Table 1, which adheres to the policy

A complete data availability statement is now provided in the manuscript, as provided here in the reporting summary.

Field-specific reporting

Please select the one below that is the best fit for your research. If you are not sure, read the appropriate sections before making your selection.

☒ Life sciences ☐ Behavioural & social sciences ☐ Ecological, evolutionary & environmental sciences

For a reference copy of the document with all sections, see nature.com/documents/nr-reporting-summary-flat.pdf

Life sciences study design

All studies must disclose on these points even when the disclosure is negative.

Sample size	Sample size was determined by the authors based on power calculations. The exact n numbers used in each experiment are indicated in the figure legends.
Data exclusions	no data were excluded from the analyses
Replication	The experimental findings were reliably reproduced as validated by at least two independent experiments. In vitro experiments were performed in triplicate whenever possible
Randomization	For in vivo experiments, animals were assigned randomly to the experimental and control groups<br>For experiments other than in vivo animal experiments, the samples were randomly allocated into experimental groups.
Blinding	Animal allocation, data acquisition and data analysis in vivo or in vitro were performed in a blinded manner.<br>For experiments other than animal experiments, the investigators were blinded to group allocation during data analysis.

Reporting for specific materials, systems and methods

We require information from authors about some types of materials, experimental systems and methods used in many studies. Here, indicate whether each material, system or method listed is relevant to your study. If you are not sure if a list item applies to your research, read the appropriate section before selecting a response.

Materials & experimental systems

n/a	Involved in the study	
☐	☒	Antibodies
☐	☒	Eukaryotic cell lines
☒	☐	Palaeontology and archaeology
☐	☒	Animals and other organisms
☐	☒	Human research participants
☒	☐	Clinical data
☒	☐	Dual use research of concern

Methods

n/a	Involved in the study	
☒	☐	ChIP-seq
☐	☒	Flow cytometry
☒	☐	MRI-based neuroimaging

Antibodies

Antibodies used	CD16/CD32 Monoclonal Antibody (FcR Block) Thermo Fisher 93 14-0161-85; Validation: C57BL/KaLwRijHsd and CD45.1+ B6.SJL splenocytes and bone marrow cells; Dilution: 1:200<br>Brilliant Violet 605™ anti-mouse/human CD11b Antibody BioLegend M1/70 101237; Validation: C57BL/KaLwRijHsd bone marrow

cells; Dilution: 1:200
APC anti-mouse CD115 (CSF-1R) Antibody BioLegend AFS98 135510; Validation: C57BL/KaLwRijHsd bone marrow cells; Dilution: 1:200
PE anti-mouse CD117 (c-Kit) Antibody BioLegend 2B8 105808; Validation: C57BL/KaLwRijHsd bone marrow cells; Dilution: 1:200
Alexa Fluor® 700 anti-mouse/human CD45R/B220 Antibody BioLegend RA3-6B2 103232; Validation:C57BL/KaLwRijHsd bone marrow cells; Dilution: 1:200
Brilliant Violet 421™ anti-mouse CD3 Antibody BioLegend 17A2 100227; Validation: C57BL/KaLwRijHsd bone marrow cells; Dilution: 1:200
Pacific Blue™ anti-mouse CD45.1 Antibody BioLegend A20 110722; Validation: CD45.1+ B6.SJL bone marrow cells; Dilution: 1:100
Alexa Fluor® 700 anti-mouse CD45.2 Antibody BioLegend 104 109821; Validation: CD45.1+ B6.SJL bone marrow cells; Dilution: 1:100
CD11b Monoclonal Antibody, APC eBioscience M1/70 17-0112-82; Validation: CD45.1+ B6.SJL bone marrow cells; Dilution: 1:200
CD11c Monoclonal Antibody, FITC eBioscience N418 11-0114-82; Validation: CD45.1+ B6.SJL bone marrow cells; Dilution: 1:200

LC3 Sigma-Aldrich Polyclonal L8918; Validation: RAW264.7 cell; Dilution: 1:1000
TRAF3 Cell Signaling Polyclonal 4729; Validation: C57BL/6J bone marrow cells and RAW264.7 cell; Dilution: 1:1000
P62 (SQSTM1) MBL Polyclonal PM045; Validation: RAW264.7 cell; Dilution: 1:1000
TRAF6 abcam Monoclonal ab33915; Validation: C57BL/6J bone marrow cells; Dilution: 1:1000
IkBα Cell Signaling 44D4 4812S; Validation: C57BL/6J bone marrow cells; Dilution: 1:1000
LAMP1 polyclonal abcam ab24170; Validation: RAW264.7 cell; Dilution: 1:1000
βActin Cell Signaling 8H10H10 3700; Validation: C57BL/6J bone marrow cells and RAW264.7 cell; Dilution: 1:5000

LAMP2 Santa Cruz Biotechnology H4B4 sc18822; Validation: U2OS cell; Dilution: 1:200
Anti-LAMP2 antibody abcam GL2A7 ab13524; Validation: RAW264.7 cell; Dilution: 1:200
Cy3-AffiniPure Goat Anti-Mouse IgG (H+L), Secondary Jackson ImmunoResearch, Polyclonal, 115-165-003; Validation: U2OS cell; Dilution: 1:500
CoraLite 488-conjugated TRAF3 Monoclonal antibody Proteintech 1E3F4 CL488-66310; Validation: RAW264.7 cell; Dilution: 1:150
Alexa Fluor™ 647 chicken anti-rat IgG (H+L) Polyclonal Invitrogen A21472; Validation: RAW264.7 cell; Dilution: 1:500
DAPI Solution Thermo Scientific 62248; Validation: U2OS cell and RAW264.7 cell

Validation

Validation method is indicated in the above box. Some antibodies were validated by the manufacturer and information was extracted from their website or documents as follow:
IRDye 800CW Donkey Anti-Rabbit IgG Secondary Antibody LI-COR 926-32213 RRID AB_621848
Dilution: 1:10,000
This antibody was tested by ELISA and/or solid-phase adsorbed to ensure minimal cross-reactivity with bovine, chicken, goat, guinea pig, hamster, horse, human, mouse, rat, and sheep serum proteins. The conjugate has been specifically tested and qualified for Western blot and In-Cell Western™ assay applications. (https://www.licor.com/bio/reagents/irdye-800cw-donkey-anti-rabbit-igg-secondary-antibody)

IRDye® 680LT Donkey anti-Mouse IgG Secondary Antibody LI-COR 926-68022 RRID AB_10715072
Dilution: 1:20,000
This antibody was tested by ELISA and/or solid-phase adsorbed to ensure minimal cross-reactivity with bovine, chicken, goat, guinea pig, horse, human, rabbit, and sheep serum proteins. The conjugate has been specifically tested and qualified for Western blot applications. (https://www.licor.com/bio/reagents/irdye-680lt-donkey-anti-mouse-igg-secondary-antibody)

Zombie Aqua™ Fixable Viability Kit BioLegend NA 423102
Used in: C57BL/KaLwRijHsd and CD45.1+ B6.SJL splenocytes and bone marrow cells
Dilution: 1:400
Validation was performed through staining a mixture of live and heat-treated Jurkat cells. The live cell population is easily distinguished from the killed population, and nearly identical results were obtained using unfixed cells.

Eukaryotic cell lines

Policy information about cell lines

Cell line source(s)

5TGM1-GFP murine MM cells were provided by B.O. Oyajobi, The University of Texas Health Science Center, San Antonio.
RAW264.7 cells were purchased from ATCC.
U2OS GFP-LC3 reporter cell line and RFP-GFP-LC3 reporter cell line were used and reported by Diao et al, Nature, 2015 [520, pages 563–566 (2015)] from our laboratories. (reference 44 in the manuscript). The cells were provided by Qing Zhong (the corresponding author of the aforementioned paper), Shanghai Jiao Tong University, School of Medicine.

Authentication

RAW264.7 cells were from commercial sources and thus not subsequently validated.
5TGM1-GFP murine MM cells were verified by supernatant IgG2b ELISA
U2OS GFP-LC3 reporter cell line and RFP-GFP-LC3 reporter cell line were verified by treating the cells with 10nM BafA1 for 2 hours and the accumulation of GFP puncta and yellow puncta were observed under confocal microscope

Mycoplasma contamination

Cell lines were routinely tested for Mycoplasma contamination, all cells used in the manuscript were tested negative.

Commonly misidentified lines
(See ICLAC register)

None

Animals and other organisms

Policy information about studies involving animals; ARRIVE guidelines recommended for reporting animal research

Laboratory animals	Sex and age matched C57BL/6J mice and CD45.1+ B6.SJL mice were purchased from Charles River, UK.<br>C57BL/KaLwRijHsd mice were purchased from Harlan, The Netherlands.<br>Myeloid specific TRAF3 knockout (LysM-Cre+, Traf3fl/fl) BM cells were used for chimeric mouse generation. Brendan Boyce's lab in University of Rochester Medical Center who provided scientific advice and prepared the TRAF3 KO mouse BM cells.<br>All mice were fed and housed under specific pathogen-free conditions at Kennedy Institute of Rheumatology, University of Oxford.<br><br>Animal information in each figure:<br>Figure 1: 8-week-old female C57BL/6J mice<br>Figure 2: 8-week-old female C57BL/KaLwRijHsd mice<br>Figure 3: 4-week-old female C57BL/6J mice<br>Figure 4: 8-week-old male C57BL/KaLwRijHsd mice<br>Figure 5: 8-week-old female CD45.1+ B6.SJL mice<br>Supplementary Figure 1: 8-week-old male C57BL/6J mice<br>Supplementary Figure 2: 8-week-old male C57BL/KaLwRijHsd mice<br>Supplementary Figure 3: 4-week-old female C57BL/6J mice<br>Supplementary Figure 4: 8-week-old male C57BL/KaLwRijHsd mice<br>Supplementary Figure 5: 8-week-old C57BL/6J mice (male and female); 8-week-old female CD45.1+ B6.SJL mice<br>Supplementary Figure 6: 8-week-old C57BL/6J mice (male and female)
Wild animals	No wild animals were used.
Field-collected samples	No field collected samples in this article.
Ethics oversight	Animal experiments were undertaken under UK Home Office Project Licenses 30/3218 and 30/3388 in accordance with the UK Animal (Scientific Procedures) Act 1986.

Note that full information on the approval of the study protocol must also be provided in the manuscript.

Human research participants

Policy information about studies involving human research participants

Population characteristics	we used 9 MM patients (8 male and 1 female) with median age at 61 (41-62).<br>Among them, 1 at ISS stage 1, 4 at ISS stage 2, 1 at ISS stage 3, 3 unknown.<br>For cytogenetics, 4 with standard risk, 3 with high risk, 2 unknown.<br>For Disease response, 5 are CR/VGPR, 2 are PR/MR, 2 are PD.<br>8 of the participants developed Bony disease at time of diagnosis. 7 with spinal disease and 1 with pelvic disease.<br><br>Abbreviations: ISS Stage 1: B2 microglobulin < 3.5mg/L and Albumin>35g/L, ISS Stage 3: B2 microglobulin>5.5mg/L, ISS stage 2: patients not fulfilling criteria for Stage 1 or 3. Adverse cytogenetics defined as per IMWG criteria: [(t(4;14), t(14:16), t(14;20) 1q gain or del 17p]. Disease response defined as per IMWG criteria CR: complete response, VGPR: very good partial response, PR: partial response, MR: minimal response, PD: progressive disease. ASCT: autologous stem cell transplantation
Recruitment	BM aspirates were donated after informed consent and covered by research ethics UCL Biobank study "Biology of Myeloma" (07/Q0502/17). Details were shown in Supplementary Table 1.
Ethics oversight	BM aspirates were donated after informed consent and covered by research ethics UCL Biobank study "Biology of Myeloma" (07/Q0502/17).

Note that full information on the approval of the study protocol must also be provided in the manuscript.

Flow Cytometry

Plots

Confirm that:

☒ The axis labels state the marker and fluorochrome used (e.g. CD4-FITC).

☒ The axis scales are clearly visible. Include numbers along axes only for bottom left plot of group (a 'group' is an analysis of identical markers).

☒ All plots are contour plots with outliers or pseudocolor plots.

☒ A numerical value for number of cells or percentage (with statistics) is provided.

Methodology

Sample preparation	Mouse blood sampling was collected by tail vein bleeding using capillary blood collection tubes (16.440, Microvette® CB 300 Z). 50ul of blood from tail vein bleeding was collected and treated with Red Blood Cell Lysis Buffer for 5 minutes. After

centrifugation, the supernatant was removed and cells were stained with antibodies (Table S3) for flow cytometry.

Mouse spleens were passed through 70 µm cell strainers (542070, Greiner Bio-One) with PBS, 0.1% BSA and 2mM EDTA to obtain single-cell suspensions. After centrifugation for 3 minutes at 400g in 4°C, red blood cells were lysed with RBC Lysis Buffer (Sigma, R7757-100ML) for 5 minutes at room temperature. RBC-lysed splenocytes were washed once more with PBS, 0.1% BSA, 2mM EDTA and then used for flow cytometry experiment. Mouse bone marrow cells were collected from the femur. Bones were crushed with a mortar and pestle in PBS-0.1% BSA-2 mM EDTA and filtered through 70 µm cell strainers, then stored on ice ready to be used for flow cytometry without RBC lysis.

Cells were first stained with fixable Zombie Aqua Live/Dead staining and FcR block in PBS for 20 minutes. Samples were then topped up with PBS-0.1% BSA-2mM EDTA as a wash. After centrifugation for 3 minutes at 400g in 4°C, the samples were stained with surface marker antibodies in PBS-0.1% BSA-2mM EDTA at 4°C for another 20 minutes. The cells were washed once with PBS-0.1% BSA-2mM EDTA.

Instrument

Four-laser LSR Fortessa X-20 (BD Bioscience)

Software

Results were analyzed with FlowJo 10.2 software.

Cell population abundance

Sorting is not applicable in this paper.

Gating strategy

Detailed gating strategies of all panels were shown in the supplementary materials. In short, a board morphology gate was first set using FSC-A/SSC-A. Singlet were gated using FSC-A/FSC-H, followed by SSC-A/SSC-H. Fluoresence gates were set using unstained and fluorescence minus one controls.

☒ Tick this box to confirm that a figure exemplifying the gating strategy is provided in the Supplementary Information.
